# STAT3 signaling in ovarian cancer: a potential therapeutic target

**DOI:** 10.7150/jca.35011

**Published:** 2020-01-01

**Authors:** Renba Liang, Xishan Chen, Li Chen, Fangzhu Wan, Kaihua Chen, Yongchu Sun, Xiaodong Zhu

**Affiliations:** Department of Radiation Oncology, Guangxi Medical University Cancer Hospital and Cancer Institute of Guangxi Zhuang Autonomous Region, Nanning, Guangxi, P.R. China

**Keywords:** STAT3, ovarian cancer, tumorigenesis, inhibitors

## Abstract

Accumulating evidence has shown that Signal Transducer and Activator of Transcription 3 (STAT3) is thought to be a promising target for cancer therapy as STAT3 is frequently overexpressed in a wide range of cancer cells as well as clinical specimens, promoting tumor progression. It is widely accepted that STAT3 regulates a variety of cellular processes, such as tumor cell growth, survival, invasion, cancer stem cell-like characteristic, angiogenesis and drug-resistance. In this review, we focus on the role of STAT3 in tumorigenesis in ovarian cancer and discuss the existing inhibitors of STAT3 signaling that can be promisingly developed as the strategies for ovarian cancer therapy.

## Introduction

**Figure 1:**
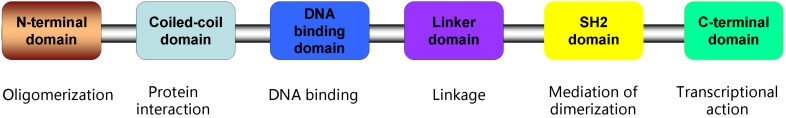
** Linear topology of STAT3 structure.** As shown, STAT3 is made up of the N-terminal domain, the coiled-coil domain, the DNA binding domain (DBD), the linker domain, the Src-homology 2(SH2) domain, and the C-terminal domain. The tyrosine residue at position 705 (Tyr-705) is close to SH2 domain.

**Figure 2:**
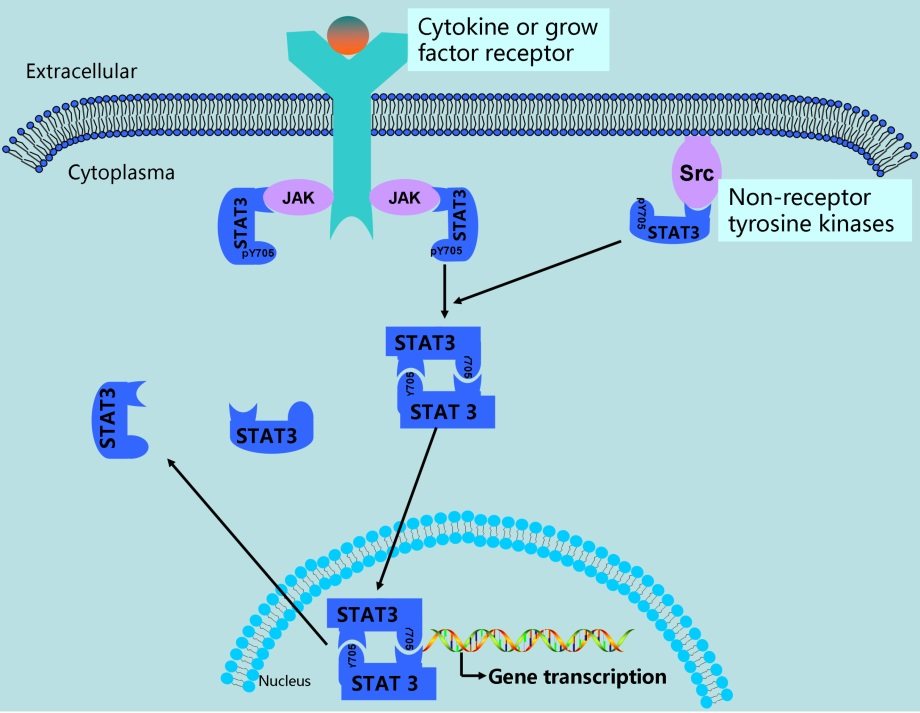
** The abnormal activation of STAT3 signaling in ovarian cancer.** In ovarian cancer, STAT3 is activated via phosphorylation of Tyr-705 by growth factor receptor tyrosine kinases, cytokine receptor associated kinases (JAK), and non-receptor kinases (Src). After activation, STAT3 forms dimerization and translocate into nucleus, in which they bind to promoter of STAT3-targeted genes, resulting in gene transcription.

Signal Transducer and Activator of Transcription 3 (STAT3) is a member of STAT family proteins which includes STAT1, STAT2, STAT3, STAT4, STAT5A, STAT5B, and STAT6^1^. Studies have identified that STAT3 consists of several distinct domains: the N-terminal domain crucial for dimer-dimer interaction, the coiled-coil containing protein interaction domain, the DNA binding domain (DBD), the linker domain, the Src-homology 2(SH2) domain binding to related receptors, the domain containing tyrosine residue at position 705 (Tyr-705), and the C-terminal domain necessary for transcriptional activation^2^-^4^(Figure 1). In general, STAT3 emerges as an inactive state located in the cytoplasm. STAT3 is activated through phosphorylation of Tyr-705 by binding to the cytoplasmic part of receptor tyrosine kinases including EGFR^5^, ^6^, or by receptor associated kinases including JAK (Janus kinase), or non-receptor kinases including Src, or diverse stimulation^4^, ^7^-^9^. Activated STAT3 forms homodimers or heterodimers through reciprocal pTyr-SH2 interaction, then translocate into nucleus and bind to special elements of STAT3-targeted genes (Figure 2), subsequently resulting in the transcription of these genes, such as Bcl2, c-myc, cyclinD1, survivin, MMP2 and MMP9^10^-^14^. Ultimately, these genes exhibit their biofunction promoting tumorigenesis and progression.

Ovarian cancer is one of the most lethal gynecological malignancies among women. According to the origin of tissue, ovarian tumor can be classified into distinct types, including epithelium cell tumor, stromal endocrine cell tumor, and germ cell tumor. Moreover, epithelial ovarian cancer (EOC), as a heterogeneous disease and accounting for over 90% of primary ovarian tumors, can also be divided into several different subtypes, such as serous, clear cell, mucinous, endometrioid, transitional cell, mixed, and undifferentiated type^15^. Unfortunately, ovarian cancer has frequently reached advanced stage when patients are at the time of diagnosis^16^, ^17^. Therefore, it is of great importance to identify the signaling pathway involved in tumorigenesis and progression of ovarian cancer.

**Figure 3:**
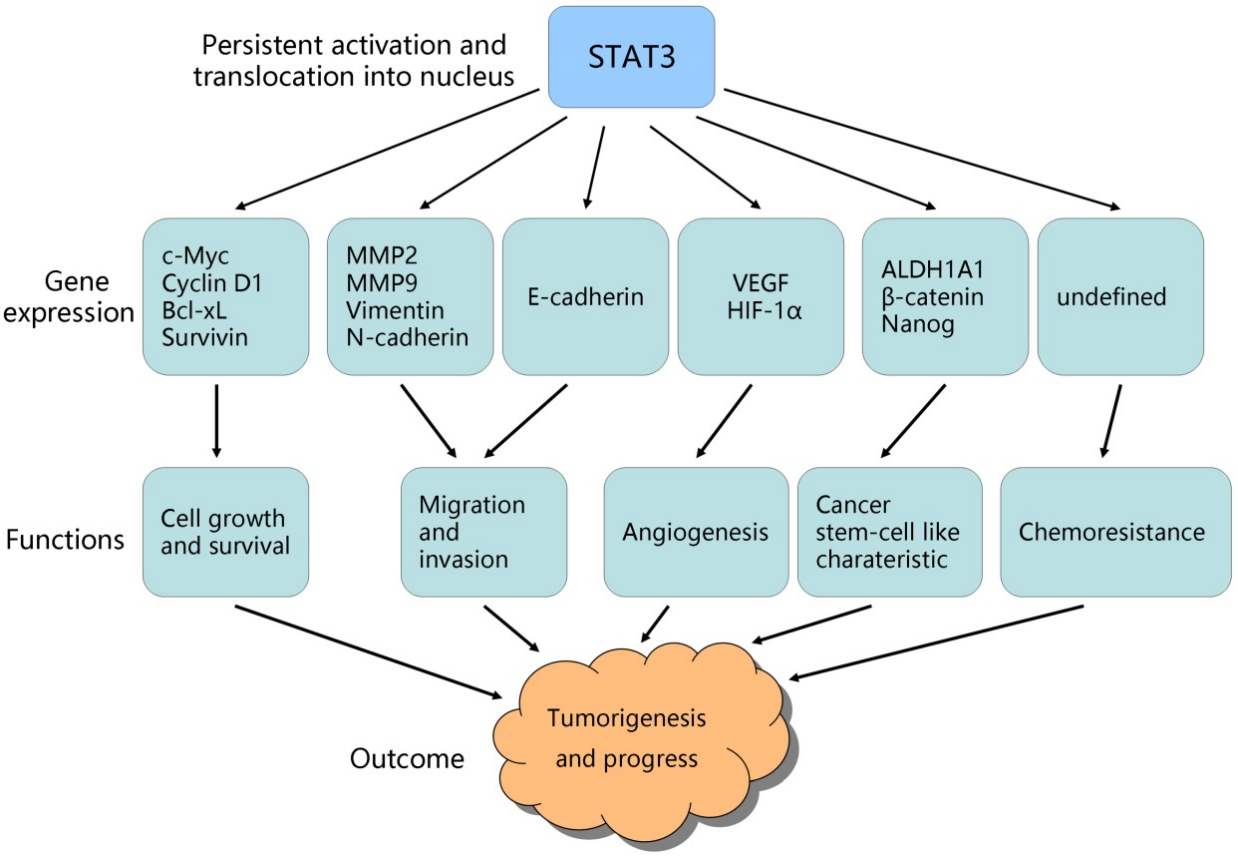
** STAT3-targeted genes and their role in tumorigenesis/progress.** Persistent activation of STAT3 promotes its-regulated genes expression, which contribute to ovarian cancer growth, survival, invasion, angiogenesis, stem cell-like characteristic, and chemo-resistance.

Interestingly, a significant body of evidence has highlighted the importance of STAT3 signaling, which is aberrantly activated in ovarian cancer cell lines and tissue samples detecting by microarray analysis, real-time reverse transcription-PCR, western blot as well as luciferase reporter, and associated with ovarian tumor development^3^, ^18^-^21^. The STAT3 signaling is critical for ovarian cancer progression, such as promoting cell proliferation, survival, invasion, stem cell-like characteristic, angiogenesis and chemo-resistance (Figure 3). Conversely, inhibition of STAT3 activation results in the dramatic suppression of tumor growth, suggesting that STAT3 signaling is a promising target for ovarian cancer therapy^22^-^25^. Thus, it is crucial to have a full understanding of functions of STAT3 in ovarian cancer in order to develop effective therapeutic interventions for ovarian cancer.

In this review, we focus on the role of STAT3 in tumorigenesis of ovarian cancer and summarize the existing agents targeting STAT3 signaling that can be potentially developed as the strategies for ovarian cancer treatment.

## Role of STAT3 in tumorigenesis in ovarian cancer

Studies demonstrates that STAT3 signaling be involved in cell proliferation, survival, invasion, stem cell-like characteristic, angiogenesis and chemoresistance in ovarian cancer:

### Migration and invasiveness

Invasion and metastasis are one of the most important characteristics of malignant tumors. Increasing number of evidence has demonstrated that STAT3 is frequently activated in ovarian carcinoma specimens, especially in high-grade type, and plays a crucial role in the migration and invasiveness of human ovarian cancer^19^, ^26^. Moreover, activated STAT3 is found located in focal adhesions known to be conducive to the motility of cell, and depletion of STAT3 decreased invasiveness of ovarian cancer cells^19^. Matrix metalloproteinase 9 (MMP9), a member of matrix metalloproteinase (MMP) family, has been widely reported to engage in the degradation of extracellular matrix, resulting in tumor invasion^27^. Interestingly, a recent study suggests that STAT3 is positively associated with expression of MMP9 in epithelial ovarian cancer. Activated STAT3 directly bind to special element of MMP9 gene promoter, inducing the increasing expression of MMP9. In addition, knockout of STAT3 decreased the expression of MMP-9 at mRNA and protein levels, which suggested that pSTAT3 may get involved in invasiveness and metastasis of ovarian cancer^28^. Likewise, alpinetin, a kind of natural flavonoid, inhibits cell migration through down-regulation of MMP‑2 and MMP‑9 via suppression of STAT3 signaling in ovarian cancer^29^. Moreover, Seo and co-workers suggest that BLT2, a leukotriene B(4) receptor, activates STAT3 and concomitantly gives rise to the overexpression of MMP2, leading to mobility of OVCAR-3 and SKOV-3 ovarian cancer cells^13^.

Epithelial-mesenchymal transition (EMT), which is marked by the downregulation of epithelial markers, such as E-cadherin, together with over-expression of mesenchymal markers, such as N-cadherin, Vimentin and snail, frequently occurs during the process of invasion and migration in tumor^30^, ^31^. It is reported that constitutively activated STAT3 is involved in EMT of ovarian cancer, as evidenced by the upregulation of Vimentin in STAT3-active cells^32^. In addition, investigations in the last decade have identified that epidermal growth factor (EGF) and its receptor (EGFR) as well as IL-6, which frequently upregulated in ovarian cancer, are crucial mediators of EMT^31^, ^33^, ^34^. Activated EGFR increases the level of N-cadherin and Vimentin, in line with activation of STAT3 and IL-6 production. Stimulating ovarian cancer cells with IL-6 promotes STAT3 phosphorylation and cell migration. Moreover, selectively blocking STAT3 signaling brings about the loss of Vimentin, N-cadherin, IL-6 as well as cell movement^26^, ^32^.

### Growth and survival

The crucial role of STAT3 in facilitating tumor cell growth and survival has been well-established^35^-^39^. At the molecular level, events at quite an early stage have indicated that constitutive activation of STAT3 has a strongly correlation with high levels of Bcl-xL, cyclin D1 and c-myc^20^, ^40^. STAT3 knockdown with specific small interfering RNA causes a loss of cell growth and induces apoptosis in human ovarian cancer cells, consistent with down-regulation of cyclin D1 and survivin level^41^. In addition, support for these finding further is provided by the evidence that treating human ovarian cancer with STAT3 inhibitor HO-3867, a novel compound which decreases the level of Tyrosine-phosphorylated STAT3 (pSTAT3) and then followed by a decline of cyclin D1, survivin and Bcl-2 as well as an ascent of cleaved PARP, caspase-3 and caspase-7, gives rise to suppression of cell proliferation and survival^42^-^44^ . Similarly, SD-1029 or SD-1008, a small molecule against JAK, induces apoptosis of ovarian cancer cells by cut-down of Bcl-X(L) and survivin expression through inhibition of STAT3 phosphorylation^45^, ^46^.

### Angiogenesis

It has been proved that angiogenesis, a process pivotal for nutrition supply for tumor growth and metastasis, is a common phenomenon in malignant disease^47^. Vascular endothelial growth factor (VEGF) and hypoxia-inducible factor-1α (HIF-1α) are two key molecules in promoting angiogenesis^48^-^51^. It is interesting to note that STAT3 regulates VEGF expression, directly binding to the VEGF promoter and strengthening expression of VEGF and tumor angiogenesis^52^-^54^. Blocking STAT3 signaling with STAT3 decoy inhibits VEGF expression and decreases tumor volumes^52^, ^55^. Furthermore, comparing with non-carcinoma tissues, primary ovarian epithelial carcinoma samples have higher level of pSTAT3 and VEGF^56^. Likewise, a study has found that IL6-STAT3-HIF signaling is abnormally upregulated in ovarian clear cell cancer samples and patients with such disease achieve clinical responses when administrated with sunitinib, a angiogenesis inhibitor^57^. Beside primary ovarian cancer sample, STAT3 is also activated in ascites-derived ovarian cancer cells (ADOCCs)^58^. HO-3867 deeply inhibits vessel formation and tumor growth in orthotopic mouse model through antagonizing STAT3^58^. In addition, it is commonly accepted that cancer stem cells (CSCs) possess property of tumorigenesis including involving in angiogenesis^59^. Similar to this knowledge, a recent study suggests ovarian cancer stem-like cells (CSLCs) are capable of differentiating into endothelial cells (ECs) and forming microtube network in the presence of chemokine CCL5. Notably, CCL5 signaling activates the NF-κB and STAT3 signal pathways, then facilitating tumor angiogenesis^60^. HIF-1α is another key modulator of angiogenesis^61^. Importantly, STAT3 plays a vital role in regulating HIF-1α expression^54^, ^62^. The same results are also found in ovarian cancer cells. Treating six ovarian cancer cell lines with diindolylmethane decreases activity of cell invasion and angiogenesis through downregulating expression of HIF-1α and VEGF via targeting STAT3^39^.

### Cancer stem cell-like characteristic

Cancer stem cells (CSCs), similar to normal stem cells (SCs), have the potential of self-renewal and differentiation. Studies more than a decade ago have suggested that CSCs exist in ovarian cancer^63^, and are believed to participate in chemoresistance, recurrence and angiogenesis^63^. Interestingly, activation of STAT3 is involved in a CSC-like residual population of ovarian cancer cell after treatment with paclitaxel. Inhibiting JAK2/STAT3 pathway brings about restraint of CSC-like characteristics in paclitaxel-treated residual cells *in vitro* and a rapid decline of tumor burden *in vivo* as expected^23^. Spheroids, known as aggregates of some malignant cells, are abundant in ovarian CSCs identified by high level of ALDH1A1 and β-catenin, which are widely considered as CSC markers^67^. Moreover, recent studies have demonstrated that STAT3 correlates with spheroid formation^24^, and c-myc, previously reported to be modulated by STAT3^20^, ^40^, is found increasing in ovarian cancer spheroids^68^, ^69^. In addition, CD24 (another CSC marker) positive ovarian cancer cells promote spheroid formation and improve tumor-initiating capacity, accompanied by increased level of pSTAT3 and STAT3 target genes, also known as stem cell genes Nanog and c-myc^70^, further suggesting that STAT3 has a tight relationship with CSCs or CSC-like phenotypes^71^, ^72^.

### Induction of chemotherapy resistance

Ovarian cancer is one of leading causes of death of patients with gynecologic cancer. Systematic chemotherapy after the cytoreductive surgery is identified standard treatment of advanced ovarian cancer. Although initial response to chemotherapy agents, especially platinum and taxane, is high, most cases unfortunately become chemoresistance, resulting in disease recurrence ultimately^15^. Therefore, a great number of studies are designed to understand the underlying mechanisms by which ovarian cancer develops resistance to chemotherapeutic agents.

Emerging evidence has suggested that pSTAT3 is highly expressed in paclitaxel- and cisplatin-resistant ovarian cancer cells^21^, ^32^, ^73^, ^74^. Inhibition of STAT3 activity reverses chemoresistance and enhances chemotherapeutic drugs-induced apoptosis, accompanied by decreased level of pro-survival genes Bcl-xL, Bcl-2 and survivin^21^, ^73^, ^75^-^78^. These results are in parallel with the findings that it increases toxicity of cisplatin or paclitaxel to ovarian cancer when treated with JAK2 or STAT3 inhibitor, such as AG490^79^, ^80^, WP1066^79^, ^80^, Diindolylmethane^39^, SD-1029^45^, and SD-1008^46^. Moreover, a recent study reveals that STAT3 polymorphisms may function as an independent marker predicting a poor response to chemotherapy for patients with advanced serous EOC^82^.

Recently, investigations demonstrate that tumor microenvironment, being make up of tumor cells, mesenchymal cells and different kinds of cytokines, is involved in chemoresistance in cancer cells^83^. Carcinoma-associated fibroblasts (CAFs), known to increase chemoresistance in tumor cells^84^-^86^, are the key components of mesenchymal cells in tumor microenvironment. Importantly, CAFs protect ovarian cancer cells from cisplatin cytotoxicity through activating STAT3 signaling^76^, ^87^. In addition, CAFs secret abundant IL‑6. Strengthening EMT via IL‑6/JAK2/STAT3 pathway results in paclitaxel resistance in ovarian cancer^88^.

## Inhibitor of STAT3 signaling in ovarian cancer

**Figure 4:**
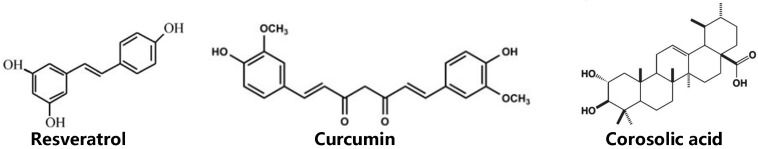
Chemical structures of natural inhibitors of STAT3 in ovarian cancer.

As discussed above, abnormal activated STAT3 plays a crucial role in tumor properties such as migration, invasiveness, proliferation, survival, angiogenesis, cancer stem cell-like characteristic, and chemoresistance in ovarian cancer, driving it to act as a promising therapeutic target to manage this kind disease. To date, a lot of approaches have been carried out around inhibiting STAT3 signaling, such as using small molecules from natural sources, synthetic agents and anti-sense oligonucleotide. Here, we will provide an outlook into natural (Figure 4) and synthetic (Table 1) inhibitors of STAT3 signaling that have been shown to be effective in ovarian cancer management.

### Natural STAT3 inhibitors in ovarian cancer

Standard chemotherapy is indispensable part of ovarian cancer treatment. Despite high sensitivity to chemotherapy, most of patients ultimately become chemoresistance. Moreover, chemotherapy drugs not only have potential to kill cancerous cells, but also generate severe toxic side effects on normal tissues and cells. These side effects limit clinical high dose use, prolong total treatment time, or even results in treatment interruption, which has a negative impact on patient's prognosis. Hence, researchers are working hard to find low-toxic and highly effective anti-tumor drugs, and natural products are an obvious example. Natural compounds are the basis of drug discovery and design, and most of anticancer drugs originate from natural products^89^. Under the efforts of researchers, to date, several natural compounds against STAT3 signaling are explored in preclinical trial or clinical trial in ovarian cancer.

#### Resveratrol

Resveratrol, a natural compound with antioxidant and anti-inflammatory function, is derived from red grapes and berries and has drew people's great attention. Resveratrol is thought to be preventive agent of cardiovascular disease^90^. There is also growing evidence that resveratrol possesses anti-tumor potential in a great number of cancers, including ovarian cancer^91^-^96^. Zhong et al found that resveratrol inhibits cell proliferation and enhances apoptosis of ovarian cancer, and activated STAT3 is the molecule target of resveratrol^37^. Similar to this finding, a recent study suggests that resveratrol suppresses growth, increases apoptosis as well as autophagic activity in ovarian cancer cells, presumably through blocking STAT3 signaling pathway^97^. In addition, IL-6, a pro-inflammatory cytokine which are able to activate STAT3^26^, promotes cell invasion and metastasis, accompanied by autophagy formation and down-regulation of ARHI (A Ras homologue member I), an imprinted tumor-suppressor gene known to suppress cell growth and motility. On the contrary, resveratrol neutralizes the effect of IL-6 on ovarian cancer cells and reduces level of STAT3 expression^95^. Furthermore, a latest report has demonstrated that ARHI is upregulated, in paralleled with decreased of STAT3 in ovarian cancer cells treated with resveratrol^98^. Altogether, preclinical practices show that resveratrol elicits antitumor effect on ovarian cancer cells. However, there is no clinical trial to assess resveratrol's property in patients with ovarian cancer. Additionally, despite phase I study conducted in patients with colorectal cancer and in healthy volunteers finds resveratrol consumed 5.0 g daily 14 days is safe and well tolerated^99^-^101^, it still warrants further clinical investigations to evaluate its clinical activity.

#### Curcumin

Curcumin (diferuloylmethane) is a phenolic component extracted from turmeric (Curcuma longa) and is often used as food additive spice. Intriguingly, modern studies have found that curcumin have more functions other than dietary spice, such as anti-inflammatory and anti-cancer effect^102^-^105^. Curcumin is involved in inhibition of transformation, survival, and metastasis in cancer^106^. For example, treating ovarian cancer cells with curcumin suppresses activation of STAT3, resulting in decreased cell viability^38^. Moreover, targeting STAT3 phosphorylation by which curcumin inhibits invasion and metastasis of ovarian cancer cells^107^. Apart from the STAT3 pathway, curcumin also inhibits characteristics of ovarian cancer via blocking the other signaling, including sarco/endoplasmic reticulum calcium ATPase, PI3K/Akt and nuclear factor-kappaB pathway^108^-^110^. Aside from its functions, it is a surprising finding that curcumin is very safe even at a high dose of oral 12 g per day in human^111^-^113^. Unfortunately, one main drawback to limit its use in medical application is rapid metabolism and low bioavailability^114^. As a consequence, many approaches have been adopted to circumvent this problem, including engineering its analogs by modulating curcumin structure^115^ and improving its delivery systems through loading in nanoparticles^116^. Take HO-3867 for example. HO-3867, a curcumin analog, exhibits substantially higher anticancer efficacy than the parent curcumin^117^. In all, improving its bioavailability, curcumin may be a great potential candidate as an anti-tumor drug in ovarian cancer.

#### Corosolic acid

Corosolic acid, a natural triterpenoid compound derived from banaba leaves and apples, has been shown to have anti-tumor effects on a variety of tumor models, such as glioblastoma^118^, prostate cancer^119^, ^120^, retinoblastoma^121^, renal carcinoma^122^, gastric cancer^123^, breast cancer^124^, liver cancer^125^, ^126^, colon cancer^127^, lung adenocarcinoma^128^, cervix adenocarcinoma^129^, osteosarcoma^130^, ^131^. Similar to curcumin and resveratrol, CA is a potent STAT3 inhibitor, suppressing cell growth of ovarian cancer and glioblastoma by abrogating STAT3 activity^118^, ^132^. Moreover, since aberrantly activated STAT3 is strongly associated with chemoresistance as mentioned above, CA also enhances cytotoxicity of chemotherapeutic drugs to ovarian cancer cells via inhibition of STAT3 activation^132^. Additionally, tumor microenvironment, as discussed previously, participates in progress of tumor^83^ and tumor-associated macrophages (TAMs) of M2 phenotype is a component of tumor microenvironment. Recent research indicates M2 macrophages regulate cell growth and metastasis of ovarian cancer^133^, ^134^, and STAT3 is activated in ovarian cancer cells when coculturing with M2 macrophages^134^. Interestingly, CA reduces STAT3 activity in ovarian cancer cells through inhibiting M2 polarization of macrophages^132^. Although the studies of CA in ovarian cancer is relatively rare comparing to curcumin and resveratrol, effects of CA have been well established in certain cancer models, providing rational evidence for conducting further investigations on it and developing it as an inhibitor in ovarian cancer.

### Synthetic blockers of STAT3 signaling in ovarian cancer

As discussed above, because of rapid metabolism or delivery systems, some natural compounds have low bioavailability in serum. Hence, it is necessary to design corresponding analogues or synthesize other novel small molecules that can inhibit STAT3 activation. Besides, given that STAT3 is made up of distinct domains and activated by receptor tyrosine kinases, or receptor associated kinases, or non-receptor kinases, or diverse cytokines, synthetic agents targeting STAT3 signaling are mainly classified into the following categories in ovarian cancer: 1) direct inhibitors targeting the domains of STAT3; 2) indirect inhibitors targeting the upstream factors.

#### Direct inhibitors of STAT3

By targeting the DNA-binding domain, N-terminal domain, SH2 domain of STAT3, direct inhibitors interfere with STAT3 activation, resulting in the blockage amplification of STAT3 signaling cascade reaction and concomitantly the decrease of STAT3-regulated gene level.

Therefore, specific domains of STAT3, in theory, are promising targets for designing STAT3 inhibitors. So far, there are some direct inhibitors which interact with the DNA-binding domain in preclinical trial of ovarian cancer. For example, HO-3867, one of diarylidenylpiperidone(DAP)-based synthetic compounds, also known as curcumin analog, directly binds to the STAT3 DNA-binding domain and exclusively inhibits activation of STAT3 without interfering with that of other member of STATs^135^. HO-3867 decreases migration of human ovarian cancer cells. Furthermore, compared with non-transformed cells and tissues, HO-3867 exerts more toxicity, including increasing apoptosis and inhibiting tumor growth, on both *in vitro* and* in vivo* using xenograft model^135^. These findings are strongly supported by the results of several reports^42^-^44^, ^58^, ^136^, ^137^. Similar to HO-3867, HO-4200 and H-4318 are two derivatives of DAP compounds that selectively interact with DNA-binding domain of STAT3. Treating cisplatin-resistant ovarian cancer cells with HO-4200 and H-4318 decreases the level of STAT3 target proteins: c-myc, Bcl-2, Bcl-xl, survivin and cyclin D1/D2, giving rise to inhibition of cell survival and induction of apoptosis. In addition, HO-4200 and H-4318 also inhibit VEGF expression and decrease migration/invasion activity^138^.

Besides DAP compounds, LC28, designed on the basis of pharmacophore of a STAT3 inhibitor inS3-54^139^, is another new inhibitor targeting the DNA-binding domain of STAT3^140^. Huang and co-workers have identified that LC28 significantly inhibits growth of cisplatin-resistant ovarian cancer cells by blocking interaction between STAT3 and DNA^140^. Beside the inhibitors mentioned above, STAT3 decoy oligodeoxynucleotides (ODN) is another strategy inhibiting DNA binding activity of STAT3^141^. The STAT3 decoy is a double-stranded oligonucleotide binding to STAT3 with a high specificity, and it exhibits anti-proliferation capacity on head and neck cancers as a phase 0 clinical trial shows^142^. Furthermore, in addition to head and neck cancers, an investigation conducted by Zhang et al has revealed that STAT3 decoy ODNs also induces cell apoptosis in xenograft mode of ovarian cancer^143^.

The SH2 domain of STAT3 is extremely important not only for recognition of related receptors, but also for dimerization of STAT3 itself or with other members of STAT family. Then STAT3 is phosphorylated and activated, subsequently translocate into nucleus to realize its biological function. Hence, the SH2 domain provides us an attractive opportunity for designing specific STAT3 inhibitors. Compared with inhibitors targeting DBD, the number of agents that inactivate SH2 domain is quite few in ovarian cancer. However, investigators still do all they can do to design new inhibitors directly acting on the SH2 domain. Stattic is an obvious example. Schust et al have suggested that Stattic, a nonpeptidic small molecule, directly inhibits the SH2 domain of STAT3, leading to inactivation of STAT3 and apoptosis of STAT3-dependent cancer cells^144^. More interestingly, as abnormally activated STAT3 plays a critical role in chemoresistance mentioned above, stattic improves the sensitivity of chemo-resistant ovarian cancer cell to cisplatin both *in vitro* and *in vivo*^73^.

Apart from DBD and SH2 domain, the N-terminal domain is a vital component of STAT3. The N-terminal domain, also called oligomerization domain, has eight helices mediating tetramerization of two STATs dimers and interaction with other proteins^145^. The forming complex may influence transcriptional activity of STAT3, indicating that the N-terminal domain of STAT3 regulates transcription of STAT3 targeted genes which are involved in tumor progress^146^. Therefore, agents targeting the N-terminal domain may potentially show anticancer efficacy. ST3-H2A2, a highly selective inhibitor of the N-terminal domain of STAT3, suppresses STAT3 signaling^147^. ST3-H2A2 induces expression of proapoptotic genes in cancer cells (PC3, DU145 prostate cancer cells and MCF-7, MDA-MB-231 breast cancer cells), but not in normal epithelial cells (prostate epithelial cells RWPE-1 and human mammary epithelial cells (HMEC)), resulting in apoptotic death of cancer cells^147^. Unfortunately, to date, there is no inhibitors of the N-terminal domain reported in the ovarian cancer.

#### Indirect inhibitors of STAT3

STAT3 signaling pathway is a cascade amplification reaction activated by either upstream kinases or diverse cytokines, such as JAK, EGFR, Src as well as IL-6. As a result, these upstream factors are attractive strategies to disturb the activation of STAT3 and there are existing several inhibitors targeting them after long years ongoing efforts. AG490 is an inhibitor of JAK2^148^. Studies have suggested that AG490 exerts anti-tumor effects on several cancers, including acute lymphoblastic leukaemia^148^, head and neck squamous cell carcinoma^149^, ovarian cancer^150^ and so on. Moreover, AG490 reverses paclitaxel resistance through decreasing the level of pSTAT3 and multidrug resistance protein 1 in ovarian cancer cells^150^. However, there is no clinical trial of AG490 in malignancies in spite of its anti-tumor efficacy in the preclinical studies. Momelotinib and ruxolitinib both are JAK inhibitors and they have been well proved to suppress ovarian cancer growth^71^, ^151^. Despite the fact that ruxolitinib is used in clinical practice in myelofibrosis and momelotinib treatment was noninferior to ruxolitinib for spleen response in Janus kinase inhibitor-naïve patients with myelofibrosis reported by a phase III randomized trial^152^, there is no clinical trial conducted in ovarian cancer. Hence, this may be next step of our work. Except for AG490, Momelotinib and ruxolitinib, there are other existing JAK inhibitors, such as AZD1480^153^, WP1066^154^, SD-1029^45^, and MLS-2384^155^ also having anti-tumor property in ovarian cancer model. It is of particular note here that although AZD1480 confers anti-tumor effects on ovarian cancer in preclinical research, few further studies have been performed in clinical. The possible reason for this phenomenon may be the severe side effect on nervous system when treating myelofibrosis with AZD1480 as a phase I clinical trial reported^156^.

Beside JAK inhibitors, the EGFR inhibitor is another way to inactivate STAT3 indirectly. Preclinical evidence reveals that EGFR inhibitors, such as Erlotinib, Cetuximab, Gefitinib and lapatinib, decrease the expression of STAT3^157^-^160^. Nevertheless, such inhibitors have minimal clinical activity or do not improve progression-free or overall survival in the treatment of patients with ovarian cancer^161^-^164^. One possible reason for this phenomenon may be the feedback activation of STAT3 signaling pathway in the long run^165^.

Src is a cell membrane-associated non-receptor tyrosine kinase and plays a critical role in proliferation, migration, and differentiation of tumor cells^166^. At the molecular level, activation of Src results in initiating of STAT3 pathway^167^. Therefore, Src is also served as an attractive therapeutic target for cancer management. In fact, a Phase II trial conducted by The Gynecologic Oncology Group has demonstrated that dasatinib, an oral Src inhibitor, shows limited efficacy in patients with recurrent epithelial ovarian cancer when administered alone^168^. However, A recent study suggests that the combination of dasatinib with paclitaxel generates synergistic inhibition in growth of ovarian granulosa cell tumor cells^169^. Interestingly, the finding of saracatinib (another Src inhibitor) is contrary to that of dasatinib as saracatinib does not improve efficacy of weekly paclitaxel in platinum-resistant ovarian cancer^170^.

IL-6 is one of the cytokines and binds specifically to its receptor (IL-6R) to form IL-6/IL-6R complex, then recruits downstream molecular gp130 and ultimately develops as a trimer IL-6/IL-6R/GP130 complex^171^. This trimer gives rise to activation of STAT3^172^. The IL-6/gp130/STAT3 signaling is frequently activated in tumors and it may be developed as a target for cancer treatment and prevention^173^, ^174^. Guo et al have reported that siltuximab, a monoclonal anti-IL-6 antibody, significantly inhibits IL-6-induced STAT3 activation and decreases the expression of STAT3 targeted gene in ovarian cancer cells. Moreover, siltuximab restores sensitivity to paclitaxel in paclitaxel-resistant ovarian cancer cell line *in vitro*. However, combination siltuximab with paclitaxel has limited effect on xenograft mouse mode *in vivo*^175^. Similarly, there is no clinical benefit from siltuximab monotherapy in patients with advanced/refractory ovarian cancer^176^. Tocilizumab is a humanized IL-6R antibody. Treating clear cell carcinoma of the ovary with tocilizumab impairs the activity of cell invasion and improves sensitivity to chemotherapy^177^. To date, a phase I clinical trial of tocilizumab is finished, finding that it is feasible and safe in EOC patients combined 8 mg/kg tocilizumab with doxorubicin or carboplatin^178^. SC144, a first-in-class orally active gp130 inhibitor, shows cytotoxicity including induction of apoptosis and cell death in ovarian cancer cells but no in normal kidney epithelial cells and endometrial epithelial cells. Furthermore, SC144 inhibits tumor growth of xenografts in mouse without substantial toxicity to normal tissues^179^.

At last, using RNA interference (RNAi) technology is another approach to block STAT3 signaling and such strategy has also been adopted in ovarian cancer cells^180^. Despite the efficacy of siRNA in cancer treatment, siRNA has not been widely used in clinical because of its instability and unsatisfactory delivery systems^181^. Hence, researchers have worked on numerous methods for overcome these carriers. Recently, an oncolytic adenovirus (M4), which selectively silences STAT3 expression by producing antisense STAT3 complementary DNA, greatly suppresses survival of ovarian cancer cells but sparing normal cells. In addition, M4 enhances cisplatin antitumor property *in vitro* and *in vivo*, and does not exert synergistic toxicity to liver when combined with cisplatin^181^. These findings provide a rationale reason for M4 further research to develop as an antitumor agent in patients with ovarian cancer.

## Conclusions

Ovarian cancer is one of leading cause of death among women. Conventional chemotherapy is a part of standard treatment in ovarian cancer. However, chemotherapy is poorly tolerated for patients as a result of severe adverse side effects and most of patients are on the road to chemoresistance. Therefore, it is urgent need to design alternative and complementary therapeutic strategies for circumventing this dilemma. Abnormally activated STAT3 has frequently been found in ovarian cancer cells and clinical specimens. Persistent activation of STAT3 enhances cell proliferation, survival, invasion, cancer stem cell-like characteristic, angiogenesis and drug-resistance in ovarian cancer. Hence, STAT3 provides us an attractive target for ovarian cancer treatment and prevention. To date, there are several natural and synthetic inhibitors targeting STAT3 signaling directly or indirectly. Some inhibitors show significant toxicity or have synergistic effects when combined with conventional chemotherapy both *in vitro* and *in vivo* but no or little, if any, on normal cells and tissues. However, in spite of great efficacy on cell lines, quite few inhibitors exhibit minimal activity in xenografts or in patients, possibly because of low bioavailability, bad delivery systems and complex environment *in vivo*. Researchers have adopted numerous ways to address these concerns. In all, given the vital role of STAT3 in progress of ovarian cancer and the published reports on STAT3 inhibitors, it is our belief that strategy targeting STAT3 signaling will achieve a great success in clinic of ovarian cancer.

## Figures and Tables

**Table 1:** Synthetic inhibitors targeting STAT3 directly or indirectly in ovarian cancer.

Agent	Target	References
HO-3867	DNA-binding domain	42-44, 58, 135-137
HO-4200	DNA-binding domain	138
H-4318	DNA-binding domain	138
LC28	DNA-binding domain	140
STAT3 ODN	DNA-binding domain	143
Stattic	SH2 domain	73
AG490	JAK2	150
Momelotinib(CYT387)	JAK2	151
Ruxolitinib	JAK2	71
AZD1480	JAK2	153
WP1066	JAK2	154
SD-1029	JAK2	45
MLS-2384	JAK**/**Src	155
Erlotinib	EGFR	160
Cetuximab	EGFR	161
Gefitinib	EGFR	162
Lapatinib	EGFR	163
Dasatinib	Src	167, 168
Saratinib	Src	169
Siltuximab	IL-6	174, 175
Tocilizumab	IL-6R	176, 177
SC144	gp130	178
siRNA-PLGA/CSO	STAT3	179
Oncolytic adenovirus (M4)	STAT3	181
